# Differential Modulation of Corticospinal Excitability by Different Current Densities of Anodal Transcranial Direct Current Stimulation

**DOI:** 10.1371/journal.pone.0072254

**Published:** 2013-08-22

**Authors:** Andisheh Bastani, Shapour Jaberzadeh

**Affiliations:** Department of Physiotherapy, School of Primary Health Care, Faculty of Medicine, Nursing and Health Sciences, Monash University, Melbourne, Australia; Katholieke Universiteit Leuven, Belgium

## Abstract

**Background:**

Novel non-invasive brain stimulation techniques such as transcranial direct current stimulation (tDCS) have been developed in recent years. TDCS-induced corticospinal excitability changes depend on two important factors current intensity and stimulation duration. Despite clinical success with existing tDCS parameters, optimal protocols are still not entirely set.

**Objective/hypothesis:**

The current study aimed to investigate the effects of four different anodal tDCS (a-tDCS) current densities on corticospinal excitability.

**Methods:**

Four current intensities of 0.3, 0.7, 1.4 and 2 mA resulting in current densities (CDs) of 0.013, 0.029, 0.058 and 0.083 mA/cm^2^ were applied on twelve right-handed (mean age 34.5±10.32 yrs) healthy individuals in different sessions at least 48 hours apart. a-tDCS was applied continuously for 10 minute, with constant active and reference electrode sizes of 24 and 35 cm^2^ respectively. The corticospinal excitability of the extensor carpi radialis muscle (ECR) was measured before and immediately after the intervention and at 10, 20 and 30 minutes thereafter.

**Results:**

Post hoc comparisons showed significant differences in corticospinal excitability changes for CDs of 0.013 mA/cm^2^ and 0.029 mA/cm^2^ (*P* = 0.003). There were no significant differences between excitability changes for the 0.013 mA/cm^2^ and 0.058 mA/cm^2^ (*P* = 0.080) or 0.013 mA/cm^2^ and 0.083 mA/cm^2^ (*P* = 0.484) conditions.

**Conclusion:**

This study found that a-tDCS with a current density of 0.013 mA/cm^2^ induces significantly larger corticospinal excitability changes than CDs of 0.029 mA/cm^2^. The implication is that might help to avoid applying unwanted amount of current to the cortical areas.

## Introduction

As part of a growing understanding of neuroplasticity, novel non-invasive brain stimulation techniques have been developed in recent years. Brain stimulation paradigms aimed at modifying corticospinal excitability include repetitive transcranial magnetic stimulation (rTMS) and transcranial electric stimulation (tES) [1], [2].

Despite the rTMS which is a neurostimulatory technique, tES is an umbrella term for description of a number of neuromodulatory techniques such as transcranial alternating current stimulation, transcranial random noise stimulation and transcranial direct current stimulation (tDCS) [2]. The most utilised techniques of tES is tDCS, application of a low-amplitude direct current which can modulate corticospinal excitability in a polarity-dependent manner [3] with several advantages. It is a painless technique with no or minimal side effects and it can be applied by an inexpensive direct current stimulator which is very simple to operate [3]. tDCS involves application of very low-amplitude direct currents (2 mA or less) via surface scalp electrodes to modify neuronal transmembrane potential and influence the level of excitability [4], [5]. Depending on the polarity of the active electrode over the primary motor cortex (M1), contralateral to the target muscles, tDCS can increase or decrease corticospinal excitability [5], [6]. Cathodal tDCS (c-tDCS) involves application of the negatively charged electrode (cathode) over M1, which leads to hyperpolarization [3], [6] of cortical neurons and reduces the size of the TMS-induced motor evoked potentials (MEPs), indicating decreased corticospinal excitability. On the other hand, anodal tDCS (a-tDCS) involves the application of the positive charged electrode (anode) over M1, which results in cortical depolarization and increases the size of TMS-induced MEPs, indicating increased corticospinal excitability [3], [6]. These changes in corticospinal excitability can lead to improved motor performances [7]–[9]; thus tDCS can be used as a stand-alone therapeutic intervention or as an add-on technique to prime the effects of other training methods [10], [11]. tDCS can also be used for induction of cortical changes to provide information about the functioning of the human brain [5].

The extent of a-tDCS-induced corticospinal excitability changes depend on the current intensity/density, the electric current per electrode surface area [3], duration of current application [3], [5], [6], [12] and the electrode’s surface area [13]. As reported in a recent systematic review [14], a-tDCS with higher current densities (CDs) induce larger corticospinal excitability changes. Nitsche and Paulus (2000) compared five current intensities between 0.2 and 1 mA (CDs between 0.006 to 0.029 mA/cm^2^). They found that a stimulus intensity of at least 0.6 mA (electrode size 35 cm^2^; CD: 0.017 mA/cm^2^) is required to induce a significant increase in MEP amplitude [3].

Although the general impression is that tDCS is a safe, well-tolerated technique with no evidence of serious adverse effects [15], [16], recipients may experience mild and transient sensory side effects such as itching, tingling and burning sensations [17]. There is a direct link between current intensity and these side effects, therefore to minimise these side effects lower intensities should be used [17], [18]. This is important, because new protocols designed to extend the duration of lasting effects recommend longer and/or multiple tDCS application sessions [2].

Despite clinical success following the application of existing tDCS parameters, involving current intensities of 1–2 mA and electrode sizes of 25–35 cm^2^ [12], stimulation parameters are yet to be optimised; more research is required to fulfil this needs. In particular, it is vital to systematically measure the effects of a range of common a-tDCS CDs on corticospinal excitability changes. Therefore, the aim of the present study was to compare the effects of a range of CDs on a-tDCS induced corticospinal excitability in healthy individuals. The second aim of this study was to assess the tolerability of a-tDCS during stimulation. We hypothesized that there is a direct relationship between the CD under the active electrode and the magnitude of induced corticospinal excitability change in M1. We also hypothesized that there is a direct relationship between CD and the level of side effects.

## Materials and Methods

### Subjects

We conducted 48 experiments on twelve healthy volunteers (seven women, five men) recruited from Monash University students/staff with a mean age of 34.5±10.3 years (age range 20–51 years), a mean weight of 68.6±11.0 kg and a mean height of 168.9±15.5 cm. All were right-handers as determined by the Edinburgh Handedness Inventory (10 item version, mean laterality quotient = 87.9±19.5) [19]. All participants completed the Adult Safety Screening Questionnaire to determine suitability for TMS [20]. Participants were informed about the experimental procedures and gave their written informed consent according to the declaration of Helsinki. All experimental procedures were approved by the Monash University Human Research Ethics Committee.

### a-tDCS of the Motor Cortex

a-tDCS was delivered by an Intelect® Advanced Therapy System (Chattanooga, USA) through a pair of saline-soaked surface sponge electrodes. The anode was placed over the left M1 for the right extensor carpi radialis muscle (ECR) as identified by TMS. The cathode was placed over the right contralateral supraorbital area [3]. The electrodes were fixed with two horizontal and perpendicular straps.

Each subject was tested at the same time of the day to avoid diurnal variations. A-tDCS was applied continuously for 10 minute for all stimulation protocols using active and reference electrodes of 24 and 35 cm^2^ respectively. A larger electrode was used for the cathode electrode to decrease the CD and reduce side effects under the indifferent electrode with more focused density under the anode [13]. The only differences between the four stimulation protocols were different current intensities (0.3, 0.7, 1.4 and 2 mA) resulting in four different CDs (D1–D4) under the active electrode (D1 = 0.013, D2 = 0.029, D3 = 0.058 and D4 = 0.083 mA/cm^2^).

### Monitoring of Corticospinal Excitability

Participants were seated upright in an adjustable podiatry chair, with the forearm pronated and the wrist joint in neutral position resting on the armrest.

Single-pulse magnetic stimuli were delivered using a Magstim 200^2^ (Magstim Company Limited, Whiteland, Wales, UK) stimulator with a flat 70 mm figure-of-eight standard magnetic coil (peak magnitude field, 2.2 T). The vertex (C_z_) point was measured and marked to be used as a reference [21]. The magnetic coil was placed over the left hemisphere (cortex), contralateral to the target muscle. The orientation of the coil was set at an angle 45° to the midline and tangential to the scalp such that the induced current flowed in a posterior-anterior direction in the brain. The area of stimulation (hotspot) was determined through the measurement of the scalp using the convention of the EEG 10/20 system to find a spot over the ECR muscle M1 that would allow measurement of the largest MEP responses.

After localizing the hot spot, the coil’s position was marked on the scalp to be used for remainder of the testing for the target muscle to ensure consistency in the placement of the coil. Resting motor threshold (RMT) was defined as the minimal stimulus intensity that evoked five MEPs in a series of 10 with an amplitude of at least 50 µV [22]–[24]. The resting thresholds for the ECR muscle were determined by incrementing and decrementing stimulus intensity in 1–2% intervals until MEPs of at least 50 µV were elicited [3]. For all further MEP measurements, the test TMS intensity was set at 120% of each individual’s RMT. Fifteen stimuli were elicited to assess corticospinal excitability at each time point. The stimulus intensity remained constant throughout the study session for each subject.

Surface EMG was recorded from the right ECR muscle using bipolar Ag/AgCl disposable surface electrodes with an inter-electrode distance of 3 cm (measured from the centres of the electrodes). To ensure good surface contact and reduce skin resistance, a standard skin preparation procedure of cleaning and abrading was performed for each electrode site [21], [25], [26]. The location of ECR was determined based on anatomical landmarks [27] and also observation of muscle response in the testing position (wrist extension and radial deviation) [28]. The accuracy of EMG electrode placement was verified by asking the subject to contract the muscle(s) of interest while the investigator monitored online EMG activity. A ground electrode was placed ipsilaterally on the styloid process of the ulnar bone [29], [30]. The electrodes were secured by hypoallergenic tape (Micropore, USA). All raw EMG signals were band pass filtered (10–1000 Hz), amplified (×1000) and sampled at 2000 Hz and collected on a PC running commercially-available software (Chart™ software, ADinstrument, Australia) via a laboratory analogue-digital interface (The PowerLab 8/30, ADinstrument, Australia). Peak-peak MEP amplitude was detected and measured automatically using a custom-designed macro in Powerlab 8/30 software after each magnetic stimulus.

### Assessment of a-tDCS Tolerability

a-tDCS side effects were assessed by monitoring the presence of itching, tingling, burning sensation and any other discomfort, including headache; these are the sensory complaints most commonly reported during application of tDCS [5], [31]. Tolerability and sensory changes were monitored based on participants’ reports under the active and/or reference electrodes at the beginning, in the middle and at the end of a-tDCS application, using numeric analogue scales (NAS) (eg, 0 = no tingling to 10 = worst tingling imaginable).

### Experimental Procedures

**Figure 1:**
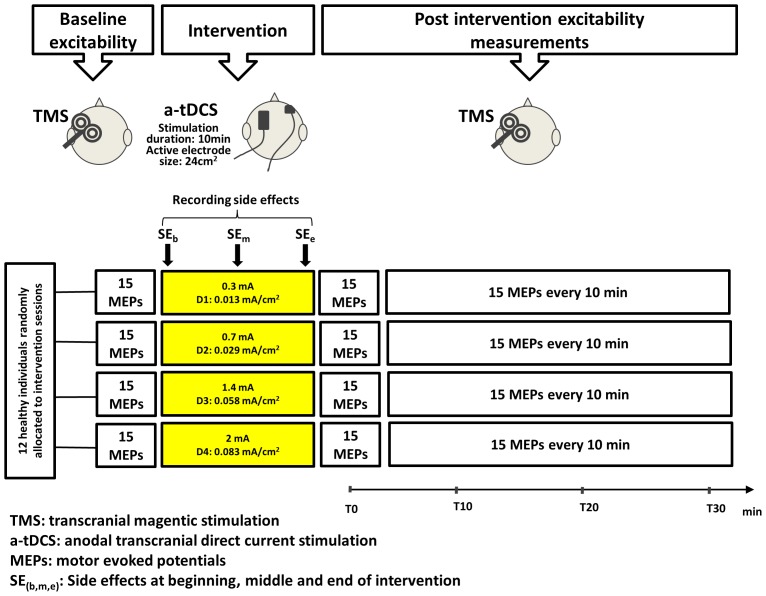
Experimental design. Comparison of the effects of different CDs (D1–D4) on corticospinal excitability.

The study was conducted in a within-subject, randomised, counter-balanced cross-over design, illustrated in Figure 1. All recruited individuals participated in four experimental sessions at least 48 hours apart to avoid interference or carry-over effects of a-tDCS. Subjects were blinded to a-tDCS conditions. The order in which the experimental sessions were conducted was randomized between participants. Corticospinal excitability was measured before, immediately after (T0) and three more times at 10-minute intervals (T10, T20 and T30) after the cessation of a-tDCS.

### Data Management and Statistical Analysis

Peak–peak amplitudes of 15 MEPs were calculated and averaged automatically for each time point before and after interventions. Post-intervention values were then normalized to the baseline value [32].

Differences in MEP amplitudes in the ECR muscle for four different a-tDCS CDs and at each of time points were analysed with a two-way repeated measure analysis of variance (ANOVA). The first within - subject independent factor was CD (four levels). The second independent factor was time points (four levels). Mauchly’s test was used to assess the validity of the sphericity assumption for repeated measures ANOVA; it requires that the variances for each set of difference scores be equal. Greenhouse-Geisser corrected significance values were used when sphericity was lacking [33]. In case of significant main effects, post hoc comparisons were performed using the least significant difference adjustment for multiple comparisons. Baseline MEP amplitudes and RMT of the respective a-tDCS conditions were tested using one-way ANOVA to see whether they were identical in all conditions. Furthermore, using one way ANOVA, we examined whether our results were associated with an order effect. We considered the results of all statistical analyses significant at *P*<0.05. All results are expressed as the mean ± standard error of mean (SEM). Statistical analyses were performed using SPSS software version 20.

## Results

### Effects of Different CDs on Corticospinal Excitability

One-way repeated measure ANOVA showed that baseline a-tDCS MEP amplitudes (*P = *0.12) and RMT were identical between all conditions (*P* = 0.28). Also, there was no significant order effect (F (3, 33) = 2.07, *P* = 0.12). Mauchly’s test of sphericity indicated that this assumption was met for CD (W = 0.387, df = 5, *P* = 0.102), so no corrections were applied to the F-ratio computations. The assumption of sphericity was violated for time (W = 0.318, df = 5, *P* = 0.05) and CD × time interaction (W = 0.000, df = 44, *P*<0.001), so Greenhouse-Geisser correction was employed for the F-ratio computations.

The results of the two-way repeated measures ANOVA showed significant main effects of time (F(1.75,19.31) = 94.05; *P*<0.001, η_p_^2^ = 0.56). Post hoc comparisons showed that there was significant difference between T0–T10 (Mean = 9.16, SE = 4.07) (*P* = 0.046), T0–T20 (Mean = 21.10, SE = 6.37) (*P* = 0.007), T0–T30 (Mean = 27.80, SE = 5.76) (*P* = 0.001), T10–T20 (Mean = 11.94, SE = 3.58) (*P* = 0.007) and T10–T30 (Mean = 18.67, SE = 3.80) (*P*<0.001). Post-hoc comparisons also indicated that there was no significant difference in the scores of T20 and T30 (*P = *0.063).

**Figure 2:**
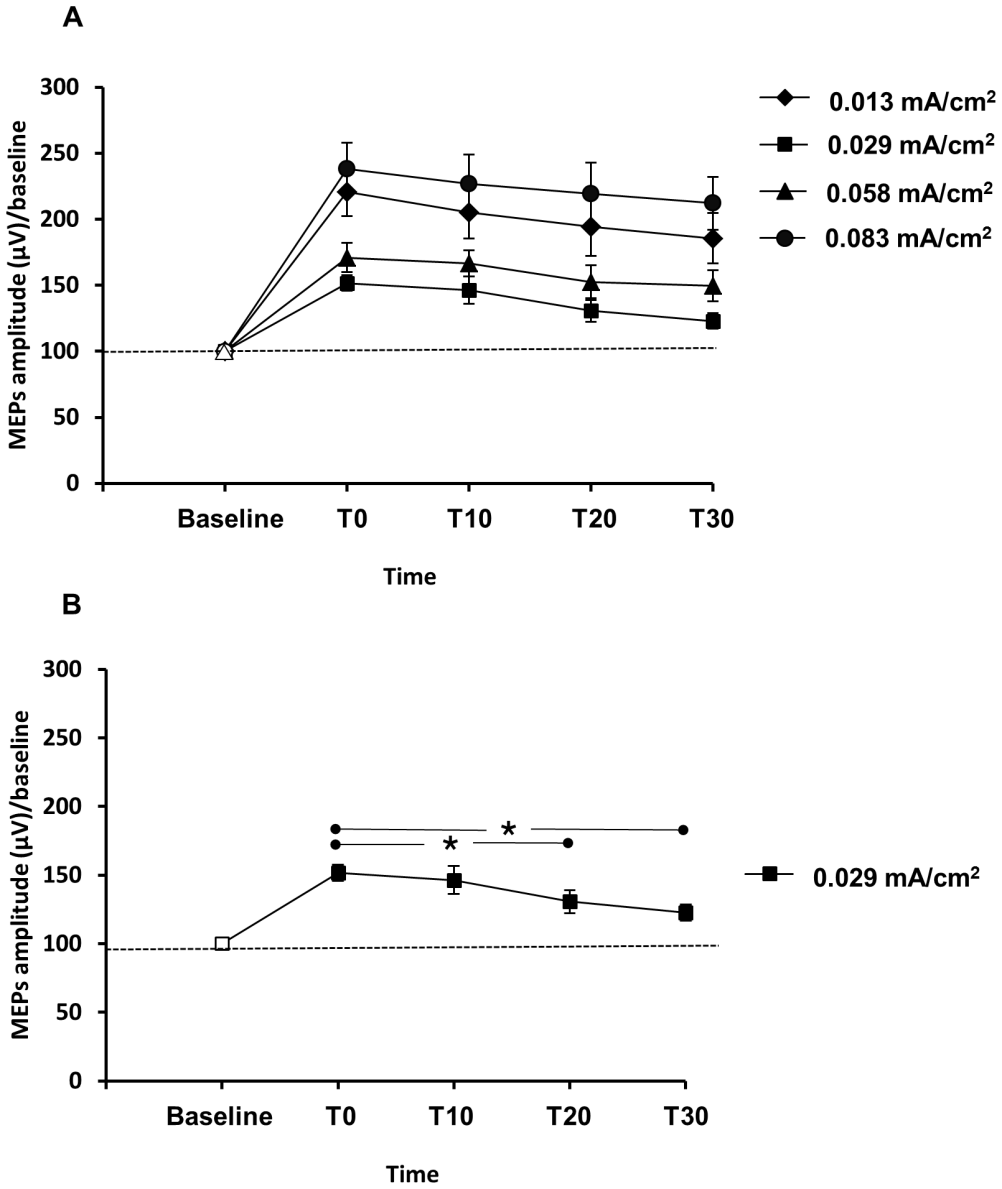
The effects of different CDs on the MEPs size over the 30 minutes. Filled symbols indicate significant deviation of the post-a-tDCS MEP amplitudes compared to baseline (A, B). The asterisks mark significant differences between time points during the 30 minutes after cessation of a-tDCS (B). The only significant differences were seen within D2 condition. Error bars represent SEM.

**Figure 3:**
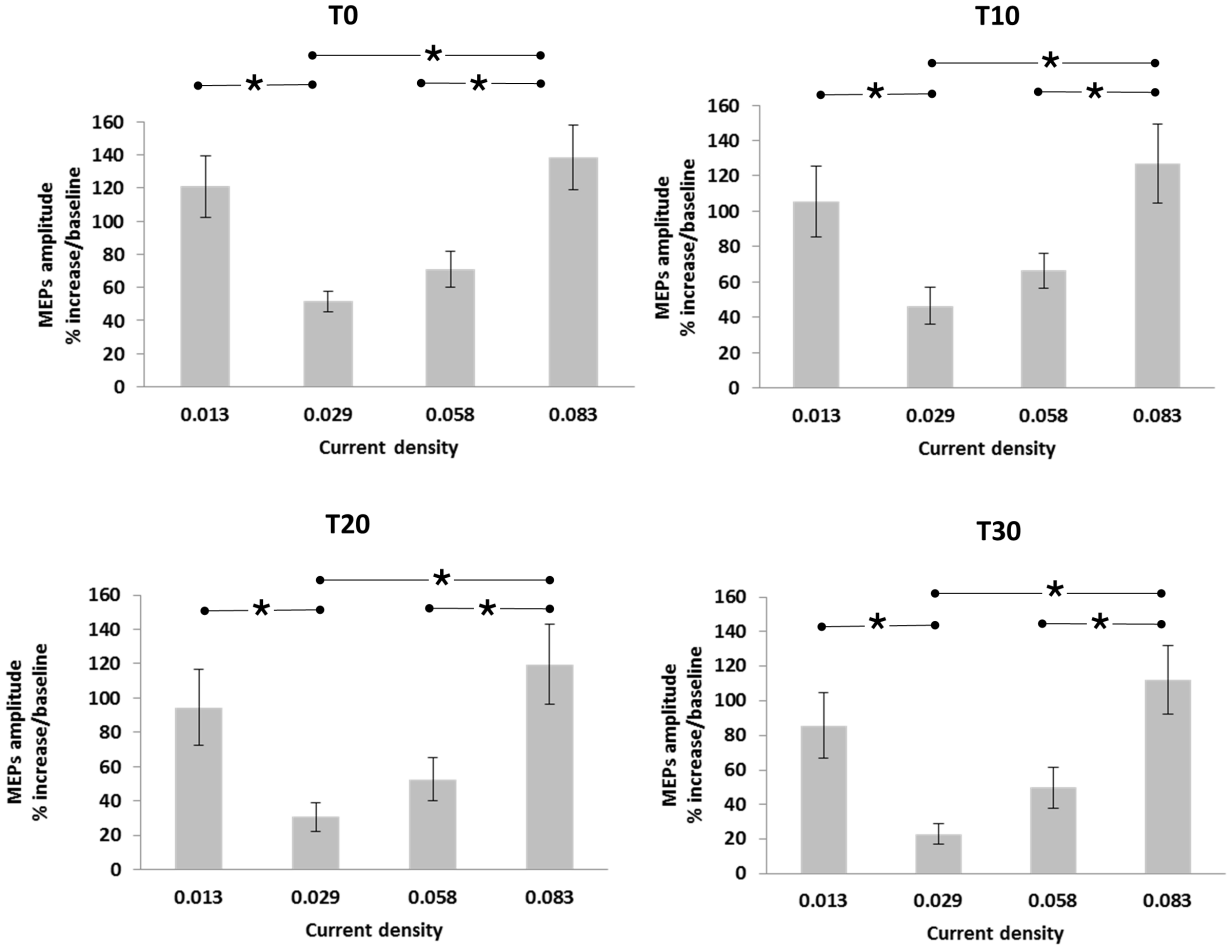
Percentage increase in corticospinal excitability after the intervention. The asterisks mark significant differences between ECR muscle MEP amplitudes after the end of a-tDCS in all time points of T0, T10, T20 and T30. Error bars represent SEM.

We observed no significant changes between different time points of a-tDCS within each D1, D3 and D4 CD conditions (*P*>0.05). However, in the D2 condition, we found significant differences between the amplitudes of ECR MEPs 20 and 30 minutes after the end of stimulation (*P*<0.05) (Figure 2). Also the result of post hoc comparisons showed significant differences between D1–D2, D2–D4 and D3–D4 (*P*<0.05) in all time points of T0, T10, T20 and T30 (Figure 3).

Two-way repeated measures ANOVA showed significant main effects of a-tDCS different CDs (F(3,33) = 6.121; *P*<0.05, η_p_^2^ = 0.36). Post-hoc comparisons indicated that there was a significant difference in the scores of D1 and D2 (Mean = 50.94, SE = 17.34) (*P*<0.05). Pairwise comparison indicated that there was a significant difference in the scores of D2–D4 (Mean = 69.04, SE = 18.05) (*P* = 0.003) and in the scores of D3–D4 (Mean = 51.38, SE = 16.97) (*P* = 0.012). Post hoc comparisons also showed that there was no significant difference between D1–D3 (*P* = 0.080), D1–D4 (*P* = 0.484) and D2–D3 (*P = *0.076) (Figure 3).

As displayed in Figure 2, a-tDCS resulted in significant excitability enhancement lasting for 30 minutes after the end of stimulation in all conditions (*P*<0.005). Finally, The results of the two-way repeated measures ANOVA showed no significant interaction of CD × time (F(3.17,34.90) = 0.18; *P* = 0.91, η_p_^2^ = 0.01). This means that the effect of CD on corticospinal excitability is not dependent on the levels of the time (T0–T30).

### a-tDCS Side Effects and Tolerability

Participants described their experiences under the electrodes at the beginning, in the middle and at the end of the intervention. The only sensations related to the cathode electrode were a mild redness under the cathode electrode (reported by two participants). In contrast, most participants reported tingling, itching and/or burning under the anode electrode (Table 1). Overall, the findings support the tolerability of direct current stimulation using CDs of D1 and D2 compared to D3 and D4. D3 and D4 produced more unpleasant feelings under the anode and the D4 caused one participant to terminate the experiment (Note that the CDs used in this study were specifically selected to allow safe stimulation). There were no adverse effects related to application of a-tDCS during the follow-up period.

**Table 1:** Sensations under the anode reported by participants.

Current Density	No sensation	Tingling sensation			Itching sensation			Burning sensation			Not tolerated
		Beginning	Middle	End	Beginning	Middle	End	Beginning	Middle	End	
D1	83.3% (10)	16.6% (2)	–	–	–	–	8.3% (1)	–	–	–	–
D2	50% (6)	50% (6)	25% (3)	8.3% (1)	–	25.0% (3)	50.0% (6)	–	–	–	–
D3	8.3% (1)	50% (6)	33.3% (4)	33.3% (4)	46.6% (5)	46.6% (5)	58.3% (7)	–	8.3% (1)	16.6% (2)	–
D4	8.3% (1)	66.6% (8)	66.6% (8)	58.3% (7)	50% (6)	66.6% (8)	66.6% (8)	16.6% (2)	25% (3)	25% (3)	8.3% (1)

The values are showed as percentage followed by number of subjects in parentheses.

## Discussion

### Effects of Different CDs on Corticospinal Excitability

The present study was designed to determine the effects of four different CDs on corticospinal excitability in healthy individuals and generated several important findings. First, different CDs induce different corticospinal excitability changes. Second, there was a direct relationship between the density of the three largest applied currents (D2, D3 and D4) and the size of the excitability changes produced. Third, in apparent contradiction to the dose-response relationship implied by the previous finding, the lowest density (D1) induced more corticospinal changes than two higher applied intensities (D2 and D3). Fourth, a-tDCS applied to the M1 increased corticospinal excitability for at least 30 minutes after the stimulation period.

We hypothesized that there is a direct relationship between the size of CDs under the active electrode and the size of induced corticospinal excitability changes in M1. The findings in the current study only support this hypothesis in part. The hypothesized direct relationship was only observed in the three largest CDs (D2, D3 and D4), but supports Nitsche and Paulus’ (2000), finding of a direct relationship between current intensities/densities of 0.2 to 1 mA (CD = 0.006 to 0.029) and corticospinal excitability changes [3]. The finding that the smallest CD produced significantly larger corticospinal changes than the next two higher CDs has not been previously reported. The finding appears to be new. However, some possible differences between the presented study and the Nitsche and Paulus (2000) study can be explained.

The findings in current study are not in line with the findings of Nitsche and Paulus (2000). Contrary to the finding in current study which indicates that the smallest CD (0.3 mA) produced significantly larger corticospinal changes than the next two higher CDs, they found that for a-tDCS, a minimal stimulus intensity of 0.6 mA (0.017 mA/cm^2^) is necessary to enhance corticospinal excitability. This discrepancy could be easily described by following differences between these two studies. First, the stimulation duration in Nitsche and Paulus (2000) study was considerably shorter than that of the current study. The stimulation time in Nitsche and Paulus (2000) study was 5 minutes compared to 10 minutes in the current study. The minimal stimulus of 0.6 may be right for 5 minutes of stimulation but that threshold should be less for longer applications. Second, Nitsche and Paulus (2000) used an electrode size of 35 cm^2^ compared to 24 cm^2^ in the current study. According to a recent study by our group [34], the electrode size has an important role on the size of induced corticospinal excitability. The electrode size of 24 cm^2^ used in our study may also contribute to the discrepancy in results with Nitsche and Paulus (2000) study.

The mechanisms underlying these changes are not clear, but it is proposed that they are caused by alterations in the function of the membrane ion channels, leading to neuroplasticity [5]. The way that a single session of a-tDCS behaves could be due to short term potentiation (STP) [35] and/or early long term potentiation (e-LTP) [36]. e-LTP depends on activation of calcium-dependent kinases, which controls the trafficking of α-amino-3-hydroxy-5-methyl-4-isoxazolepropionic acid (AMPA), and activation of N-methyl-D-aspartate (NMDA – a subtype of glutamate receptor) [37]–[41]. Excitatory synaptic changes in the brain are predominantly mediated by the neurotransmitter glutamate [42], [43], while inhibitory transmission is mediated mainly by the neurotransmitter gamma-amino butyric acid (GABA) [42], [44]. The level of excitation in the brain is kept in check through inhibitory control exerted by GABA neurons [45]. One pharmacological study showed an abolition of the intracortical effects of anodal tDCS after administration of lorazepam as a GABA agonist [46]. Also, in a recent animal study it was shown that any increase in NMDA activity coincides with an increase in the level of GABA secretion [47]. The mechanism behind this activation of GABA receptors could be that the Ca^2+^ influx through the NMDA receptors affects the adjacent inhibitory presynaptic sites and leads directly to release of GABA [48]. In addition to this, the activation of gated Ca^2+^ channels on the synaptic membrane may play a role [49], [50]. Thus, any manipulation that influences the magnitude or dynamics of Ca^2+^ increases within dendritic spines may profoundly influence the form of the resulting synaptic plasticity.

Surprisingly, we found that the smallest CD (D1) induced larger corticospinal changes than the two consecutive higher CDs of D2 and D3. This finding has no precedent in the literature; it indicates that a different mechanism may be involved in induction of corticospinal excitability changes at lower current intensities/densities. Lower density of a-tDCS may induce more corticospinal changes than higher densities due to the relative activity of facilitatory and inhibitory mechanisms. Previous animal studies have reported that GABA activation is voltage dependent [51], [52]. An increase in MEP amplitude with D1 may be due to the fact that at this low density the GABA and NMDA receptors are inactive and the excitatory changes are driven by activation of voltage-gated Ca^2+^ channels which normally have lower thresholds than NMDA or AMPA receptors. Apparently, this low direct current stimulation at 0.3 mA (0.013 mA/cm2), considered a weak form of a-tDCS, is sufficient to activate Ca^2+^ channels and raise intracellular Ca^2+^ concentrations. This may lead into cortical neuron depolarization that shifts the resting membrane potential more toward positive values and closer to the threshold level, a state called ‘excitation’.

In the current study, lasting effects of a-tDCS (increased corticospinal excitability) were measured up to 30 minutes after the end of stimulation, consistent with previous investigations [6], [53]–[56]. These observations suggest that the modulatory response of M1 pyramidal cells to a-tDCS might be dependent on the CD and subsequent degree of activated receptors.

### a-tDCS Side Effects and Tolerability

Our findings confirm that the smallest CD (0.013 mA/cm^2^) has the lowest side effects under the active electrode, thereby supporting our second hypothesis. The application of a-tDCS to the ECR M1 area was associated with a tingling sensation in 40.8% of the tests in all CD conditions, however; 25.0% of recipients of D3 and 66.7% of the participants who received D4 found the stimulation procedure mildly unpleasant.

### Limitations

Our findings must be interpreted in the context of several limitations. First, our study involved only 12 non-randomly-selected participants, which limits the generalizability of the results. The data were obtained from a healthy population, so we cannot extrapolate the findings to patient populations. The effects of the stimulation were only assessed up to 30 minutes after delivery; longer assessment of lasting effects is recommended to evaluate their length. Another limiting factor is that the examiner was not blinded to the stimulation conditions.

### Suggestions for Future Studies

A further study involving current intensities between 0.3–0.8 mA is suggested to investigate the turning point of the excitability changes. Furthermore, to underpin the mechanisms of action of lower CD, it is recommended that a study of motor cortex excitability be undertaken, by measuring silent period, intracortical inhibition, and facilitation, to indirectly assess the role of GABAa, GABAb and glutamergic receptors.

In addition, the effects of different CDs and their tolerability should be studied in patients with neurological problems, different age groups and genders. Additional pharmacological experiments using receptor agonists/antagonists are needed to prove that if a-tDCS with lower CD has different mechanisms compared with larger CD.

## Conclusion

Our findings can be employed to develop a-tDCS protocols optimized for clinical application. The smallest CD used in this study (0.013 mA/cm^2^) could be a promising parameter for the modulation of corticospinal excitability with less total charge to the cortical area. In addition to its efficiency in inducing corticospinal excitability, it was much better tolerated than larger CDs and could be safely used in protocols with multi sessions of a-tDCS applications. Our results suggest that a deeper understanding of the mechanisms underlying a-tDCS-induced excitability is required.
